# Perioperative management with DMARDs in rheumatic diseases: a scoping review of clinical guidelines

**DOI:** 10.1186/s41927-025-00522-x

**Published:** 2025-07-03

**Authors:** Alice Terrett, Athena Chin, Mihye Kwon, Samuel Whittle, Catherine Hill

**Affiliations:** 1 Rheumatology Unit, The Queen Elizabeth Hospital, Woodville South, South Australia Australia; 2 Rheumatology Unit, Royal Adelaide Hospital, Adelaide, South Australia Australia; 3 Department of Internal Medicine, Konyang University Hospital, Daejeon, South Korea; 4 Faculty of Health and Medical Sciences, University of Adelaide, Adelaide, South Australia Australia

**Keywords:** Rheumatoid arthritis, Ankylosing spondylitis, Systemic lupus erythematosus, DMARDs

## Abstract

**Objective:**

Patients with autoimmune rheumatic diseases have high rates of surgical procedures including joint replacements despite the use of disease-modifying anti-rheumatic drugs (DMARDs). This scoping review compares clinical practice guideline recommendations for the perioperative management of DMARDs in such patients.

**Methods:**

Medline and EMBASE were searched, and a hand search of references was performed to obtain guidelines published since 2014 by national/international academic societies in rheumatology addressing perioperative management of DMARDs in any of adult rheumatoid arthritis (RA), ankylosing spondylitis (AS), psoriatic arthritis (PsA), juvenile idiopathic arthritis (JIA) or systemic lupus erythematosus (SLE). Data extraction was performed in duplicate by two authors.

**Results:**

Twelve guidelines were included − 10 (83%) incorporated a perioperative recommendation within a broader guideline. RA was the sole rheumatic condition in 6 (50%) guidelines. Low-moderate quality evidence supported these recommendations, based on evidence from studies of participants undergoing elective orthopaedic surgery. Guidelines varied in development process, format, the choice of evidence system, level of evidence, strength of recommendation and recommendations for biologic DMARD (bDMARD) use and timing of surgery.

**Conclusion:**

Although guidelines for the use of DMARDs in the perioperative period are widely available, the development process and recommendations vary between guidelines. There is a lack of high quality evidence to support recommendations for non-elective, non-orthopaedic surgery cases. Variations in recommendations for bDMARDs in the perioperative period were common, potentially leading to more practice variation in bDMARD use in the perioperative period. Continued accrual and review of evidence will provide greater support for recommendations in this clinical setting.

**Supplementary Information:**

The online version contains supplementary material available at 10.1186/s41927-025-00522-x.

## Background

The treatment paradigm of autoimmune rheumatic diseases (including rheumatoid arthritis (RA), spondyloarthritis (SpA), psoriatic arthritis (PsA), ankylosing spondylitis (AS), systemic lupus erythematosus (SLE)) has evolved with the increasing availability of disease modifying anti-rheumatic drugs (DMARDs), with improvement in mortality rates, symptomatology and functional outcomes [1–3].

Whilst rates of hand and foot surgery in patients with RA have declined, rates of large joint replacements remain static [4]. Population studies have demonstrated that up to 80% of RA patients undergoing joint replacement are on methotrexate or another conventional synthetic DMARD (csDMARD) [5]. Despite improvement in functional outcomes and pain post-operatively, higher rates of adverse surgical outcomes, including infection, are seen in patients with RA or SLE undergoing arthroplasty [6, 7]. The aetiology for higher rates of infection is not specifically linked to use of DMARDs (though this has not yet been validated in randomised control trials) and could potentially relate to underlying dysregulation of the immune system in patients with autoimmune rheumatic diseases (AIRD) [8].

Concerns remain regarding the optimal method for balancing the risks and benefits of the use of DMARDs in the perioperative period. Continuation of DMARD therapy during this period may be associated with a theoretical increased risk of infection, however in contrast temporary cessation may increase the risk of a flare of the associated autoimmune rheumatic disease. The aim of this review is to compare existing clinical practice guideline recommendations for the perioperative management of DMARDs in patients with rheumatic diseases.

## Materials and methods

### Type of review

As the intention of this review was to collect available information from a range of diverse sources and identify knowledge gaps, we performed a scoping review. The review followed established methodological guidance from JBI Scoping Review Methodology Group [9]. The Preferred Reporting Items for Systematic reviews and Meta-Analyses extension for Scoping Reviews (PRISMA-ScR) Checklist is attached as supplementary file 1.

### Search strategy

In consultation with a research librarian, two main searches were performed of Medline and EMBASE databases for relevant publications (supplementary file 2). Reference lists of included guidelines were hand searched for additional guidelines. Initially, the titles and abstracts were pre-screened by a single reviewer (MK), followed by screening by two reviewers (AC, AT) with discrepancies resolved by discussion. A full text review was performed by two reviewers (AC, AT) with selection of articles based on pre-determined inclusion and exclusion criteria. The inclusion criteria were: (1) recommendations, guidelines type, published within the last 10 years (2014–2024), (2) author/group of national or international academic societies in rheumatology, (3) target disease and patients; adult RA, AS, PsA, JIA (juvenile idiopathic arthritis), or SLE, (4) target drugs; csDMARDs (methotrexate, sulfasalazine, leflunomide, hydroxychloroquine, minocycline), biologic DMARDs (bDMARDs) (adalimumab, etanercept, infliximab, golimumab, certolizumab, abatacept, tocilizumab, rituximab, sarilumab, anakinra), targeted synthetic DMARDs (tsDMARDs) (tofacitinib, baricitinib, upadacitinib, filgotinib). Glucocorticoids (including prednisolone) were not included as a target drug. The exclusion criteria were: (1) systematic reviews/scoping reviews, meta-analyses, observational studies, RCTs, (2) author/group of individual hospitals or research groups, (3) target drugs; publications exclusively focused on immunosuppressants, (4) no English translation available. In the event of two guidelines published by the same specialty society in the review timeframe, the most recent guideline was included for relevance.

### Data extraction

Demographic data were extracted for each guideline by two authors (AC, AT), including year of publication, country/region of publication, included rheumatic conditions, conflict of interest declaration and management, panel structure, method of formulation of recommendation, evidence system used and included DMARDs. Recommendations regarding the perioperative management of DMARDs were extracted. In-text recommendations were not extracted. During data extraction, the items to be extracted were further refined through discussions between the two researchers. A detailed list of categories of data extracted is included in supplementary file 3.

## Results

### Literature search

**Fig. 1:**
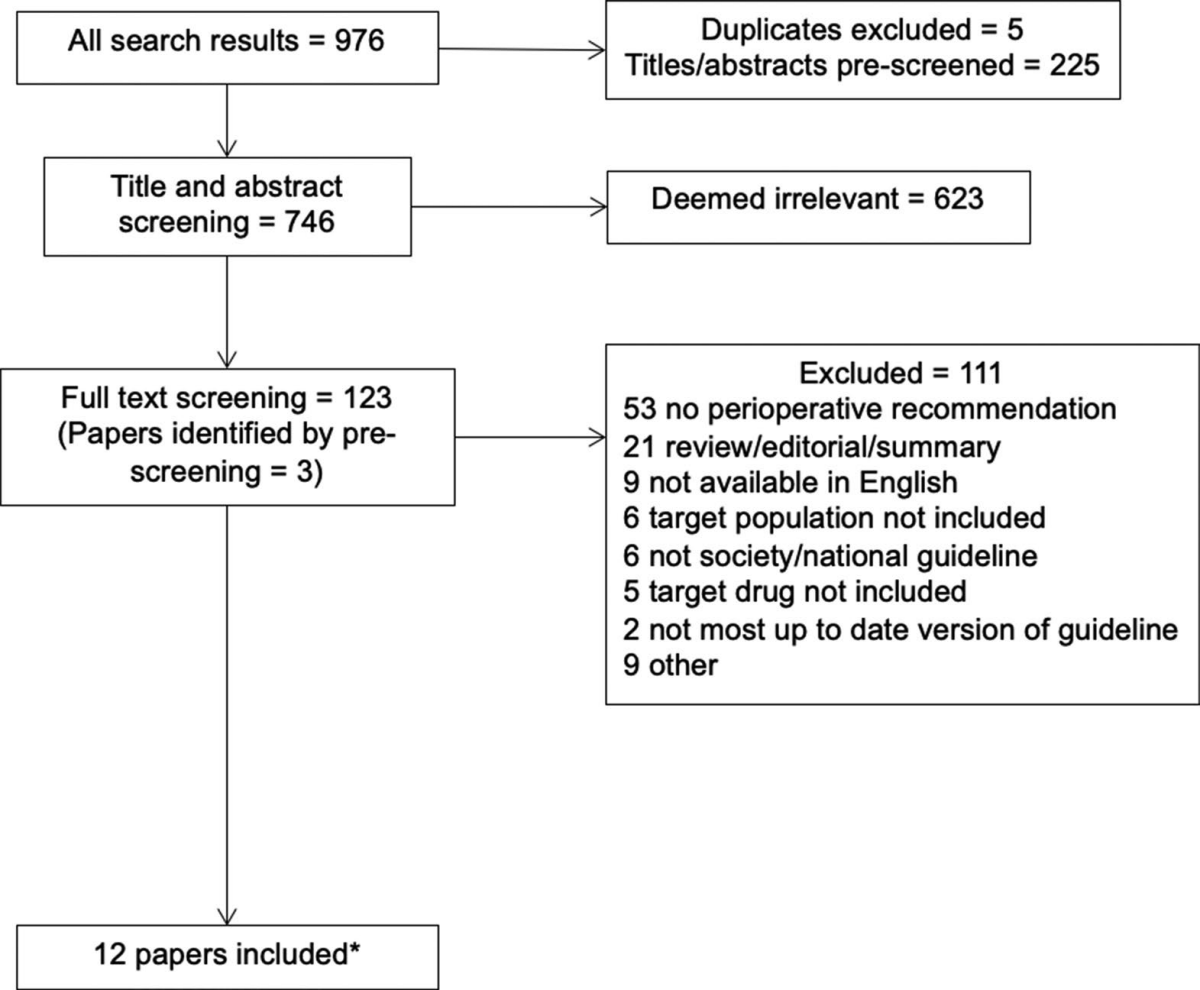
PRISMA flowchart

The above search strategy identified 971 unique articles. After title and abstract screening, 120 articles were selected for full text review. An additional 3 articles were identified by hand search. 111 were excluded after full text review for the following reasons: no perioperative recommendation (*n* = 53), review/editorial/summary article (*n* = 21), not available in English (*n* = 9), not a national society guideline (*n* = 6), target drugs not included (*n* = 5), not the most up to date version of guideline from a specialty society (*n* = 2) and other (*n* = 9) (including: defining scope for future guidelines, comparison of guidelines, article subsequently retracted). 12 articles, with 12 corresponding guidelines, were included in this review [10–21] (Fig. 1). An abridged English translation was available for a single guideline [17]. The recommendations from this guideline were included in the review.

### Characteristics of included guidelines

The 12 included guidelines were published between 2014 and 2024 (Table 1). Guidelines predominantly originated from either European [10, 13, 14, 16, 19] (*n* = 5, 42%) or Asia-Pacific professional societies [11, 17, 18, 20, 21] (*n* = 5, 42%), with the remainder originating from North America [12, 15] (*n* = 2, 17%). Two (17%) were a standalone guideline for the use of DMARDs in the perioperative period [10, 15], the remaining 10 (83%) incorporated a recommendation into a broader guideline. RA was the sole rheumatic condition covered in 6 (50%) guidelines [12–14, 17, 18, 20]. Recommendations for csDMARDs were included in 8 (67%) [10–15, 17, 19], bDMARDs in 8 (67%) [10–12, 15, 16, 18, 20, 21], and tsDMARDs in 5 (42%) [10, 11, 15, 18, 20]. In addition, 3 (25%) guidelines also provided a recommendation for glucocorticoids [10, 11, 19] and 3 (25%) provided a recommendation for drugs not included in our inclusion criteria (apremilast, mycophenolate mofetil, cyclosporin A, azathioprine, tacrolimus, ustekinumab, secukinumab, guselkumab, belimumab, anifrolumab, voclosporin, ixekizumab) [10, 15, 16, 19]. Funding sources were disclosed in 6 (50%) guidelines [11, 12, 14, 18, 20, 21] and conflicts of interest from panel members in 10 (83%) [10–12, 14–16, 18–21], as outlined in Table 1.

**Table 1:** Characteristics of included guidelines

Author	Year of publication	Region	Rheumatology specialty society	Rheumatic conditions	Standalone perioperative guideline	Funding source	Conflicts of interest
Albrecht et al. [10]	2023	Europe	German Society for Rheumatology (DGRh)	RA, inflammatory diseases	Yes	Not disclosed	Declared, nil required management
Cardiel et al. [12]	2014	South America	Mexican Society of Rheumatology (CMR)	RA	No	Education grant, SANOFI	Declared, management not disclosed
Duarte et al. [13]	2017	Europe	Portuguese Society of Rheumatology (SPR)	RA	No	Not disclosed	Not declared
Garcia-Vicuna et al. [14]	2017	Europe	Spanish Rheumatology Society (SER)	RA	No	SER	Declared, management not disclosed
Glennon et al. [11]	2024	Asia-Pacific	Australia & New Zealand Musculoskeletal Clinical Trials Network (ANZMUSC), Australian Rheumatology Association (ARA)	RA, SpA, PsA	No	Grant funding*	Declared, managed via risk matrix
Goodman et al. [15]	2022	North America	American College of Rheumatology (ACR)	RA, SpA, JIA, SLE	Yes	Not disclosed	Declared, management declared
Holroyd et al. [16]	2019	Europe	British Society of Rheumatology (BSR)^#^	RA, SpA, PsA	No	Not disclosed	Declared, management not disclosed
Kameda et al. [17]	2019	Asia-Pacific	Japan College of Rheumatology (JCR)	RA	No	Not disclosed	Not disclosed
Lau et al. [18]	2019	Asia-Pacific	Asia-Pacific League of Associations for Rheumatology (APLAR)	RA	No	APLAR	Declared, management not disclosed
Ledingham et al. [19]	2017	Europe	British Society of Rheumatology (BSR)/British Health Professionals in Rheumatology (BHPR)^##^	Rheumatic disease	No	Not disclosed	Declared, management not disclosed
Louthrenoo et al. [20]	2017	Asia-Pacific	Thai Rheumatism Association (TRA)	RA	No	TRA	Declared, management not disclosed
Tsai et al. [21]	2021	Asia-Pacific	Taiwan Rheumatology Association (TRA)	PsA	No	TRA, TAPSI^^^, (submission funded by Novartis)	Declared, nil required management
Legend: RA = rheumatoid arthritis, SpA = spondyloarthritis, PsA = psoriatic arthritis, SLE = systemic lupus erythematosus, JIA = juvenile idiopathic arthritis *Australian guidelines funded by Department of Health (2019–2022), National Health and Medical Research Council (NHMRC) Australia & New Zealand Musculoskeletal (ANZMUSC) Clinical Trials Network Centre of Research Excellence (2023–2027), Cochrane MSK, Victorian Government ^#^biologic DMARD guideline ^##^non-biological DMARD guideline ^^^ Taiwanese Association for Psoriasis and Skin Immunology							

				Recommendation regarding			
Author	Rheumatology specialty society	Panel structure	Evidence system	csDMARD	bDMARD	tsDMARD	Other (including DMARDs not in inclusion criteria)
Albrecht et al. [10]	German Society for Rheumatology (DGRh)	Not specified	OCEBM	Yes	Yes	Yes	Yes (prednisolone, azathioprine, apremilast, cyclosporine A, ixekizumab)
Cardiel et al. [12]	Mexican Society of Rheumatology (CMR)	R	NICE	Yes	Yes	No	No
Duarte et al. [13]	Portuguese Society of Rheumatology (SPR)	R	OCEBM	Yes (MTX only)	No	No	No
Garcia-Vicuna et al. [14]	Spanish Rheumatology Society (SER)	R	SIGN	Yes (MTX only)	No	No	No
Glennon et al. [11]	Australia & New Zealand Musculoskeletal Clinical Trials Network (ANZMUSC), Australian Rheumatology Association (ARA)	R, Ph, I, GP, Po, C, O	GRADE	Yes	Yes	Yes	Yes (prednisolone)
Goodman et al. [15]	American College of Rheumatology (ACR)	R, O, ID, SLEe, C, M, E	GRADE	Yes	Yes	Yes	Yes (mycophenolate, azathioprine, cyclosporine, tacrolimus, ustekinumab, secukinumab, guselkumab, belimumab, anifrolumab, voclosporin, ixekizumab)
Holroyd et al. [16]	British Society of Rheumatology (BSR)^#^	R, T, N, C, A	GRADE	No	Yes	No	Yes (ustekinumab)
Kameda et al. [17]	Japan College of Rheumatology (JCR)	Not specified	Not specified	Yes (MTX only)	No	No	No
Lau et al. [18]	Asia-Pacific League of Associations for Rheumatology (APLAR)	Not specified	GRADE	No	Yes	Yes	No
Ledingham et al. [19]	British Society of Rheumatology (BSR)/British Health Professionals in Rheumatology (BHPR)^##^	R, T, N, GP, Ph, C	GRADE	Yes	No	No	Yes (glucocorticoids, mycophenolate, azathioprine, tacrolimus)
Louthrenoo et al. [20]	Thai Rheumatism Association (TRA)	Not specified	SIGN	No	Yes	Yes	No
Tsai et al. [21]	Taiwan Rheumatology Association (TRA)	R, D, O, Po	GRADE	No	Yes	No	No

Legend: R = Rheumatologists, Ph = pharmacists, I = immunologists, GP = general practitioners, Po = podiatrists, C = consumers, O = orthopaedic surgeons, ID = infectious diseases physician, SLEe = SLE expert, M = methodologists, D = dermatologists, E = GRADE expert, T = rheumatology trainee, N = nurse specialist, A = academic, OCEBM = Oxford Centre for Evidence-Based Medicine, GRADE = Grading of Recommendations, Assessment, Development, and Evaluations, NICE = National Institute for Health and Care Excellence, SIGN = Scottish Intercollegiate Guidelines Network, MTX = methotrexate

### Formulation of recommendations

The method used to generate guideline recommendations was similar between groups, following a review of literature and panel consensus discussion prior to a voting round to determine agreement with the recommendation. Panel structure varied between societies, with panel composition reported in 8 (67%) guidelines [11–16, 19, 21]. Where specified, rheumatologists were present in all panels (*n* = 8). Other panel members varied between guidelines, including orthopaedic surgeons (*n* = 3), general practitioners (*n* = 2), pharmacists (*n* = 2), immunologists (*n* = 1), nurses (*n* = 1), dermatologists (*n* = 1), and trainee rheumatologists (*n* = 1). Patient or consumer representatives were only included in 4 (33%) guidelines [11, 15, 16, 19]. A breakdown of panel structures is included in Table 1.

### Rating of evidence

The method selected to rate the level of evidence used to formulate the guidelines varied between each publication with all but one guideline specifying the evidence grading. The most commonly used methodology was Grading of Recommendations, Assessment, Development, and Evaluations (GRADE, *n* = 6, 50%) [11, 15, 16, 19, 21] followed by the Oxford Centre for Evidence-Based Medicine (OCEBM) Levels of Evidence (*n* = 2, 17%) [10, 13] and the Scottish Intercollegiate Guidelines Network (SIGN) (*n* = 2, 17%) [14, 20]. National Institute for Health and Care Excellence (NICE) was used in a single guideline [12] and the method was not specified in the remaining guideline [17].

### Recommendations regarding conventional synthetic DMARDs (csDMARDs)

Of the 8 guidelines that included a recommendation for the perioperative management of csDMARDs [10–15, 17, 19], recommendations regarding hydroxychloroquine were present in 4 (50%) [10, 11, 15, 19], sulfasalazine in 4 (50%) [10, 11, 15, 19], leflunomide in 4 (50%) [10, 11, 15, 19] and methotrexate in 8 (100%). Three (38%) only included a recommendation for methotrexate [13, 14, 17]. The strength of the recommendation was weak/conditional in 4 (50%) [11, 12, 14, 15] and unspecified in 4 (50%) [10, 13, 17, 19]. There was 100% agreement between guidelines with regard to the recommendations for hydroxychloroquine and sulfasalazine (‘do not routinely discontinue’), 75% with regard to leflunomide (1 guideline suggested management is dependent on infection risk) and 88% with regard to the recommendation for methotrexate (1 guideline suggested holding methotrexate for 1 week prior to operative intervention, the remainder suggested continuing dependent on patient factors, such as pre-operative control of their inflammatory disease, risk of infection, risk of flare, and patient preference). Overall, there was a low certainty of evidence underlying each of the recommendations, and a particular absence of evidence for recommendations in non-elective, non-orthopaedic surgery cases. There was however, a high level of agreement (70–100%) between panel members with regard to the content of the recommendations when reported in 3 of the 8 guidelines [10, 13, 14]. A summary of the recommendations for conventional synthetic DMARD use in the perioperative period is included in Table 2.

**Table 2:**
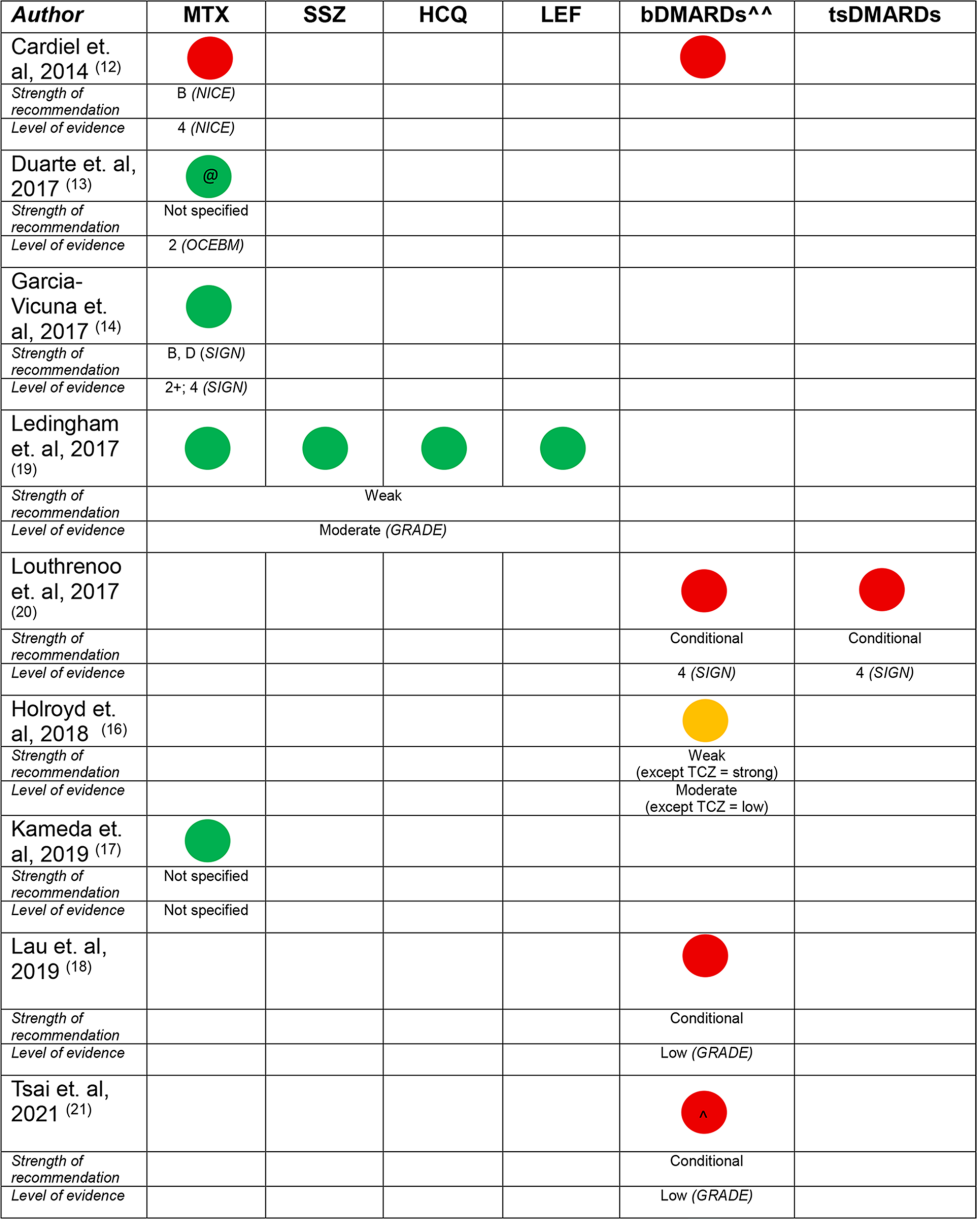
Summary of recommendations for use of conventional synthetic, biologic^^ and targeted synthetic DMARDs in the perioperative period (arranged by year of publication)

**Table:**
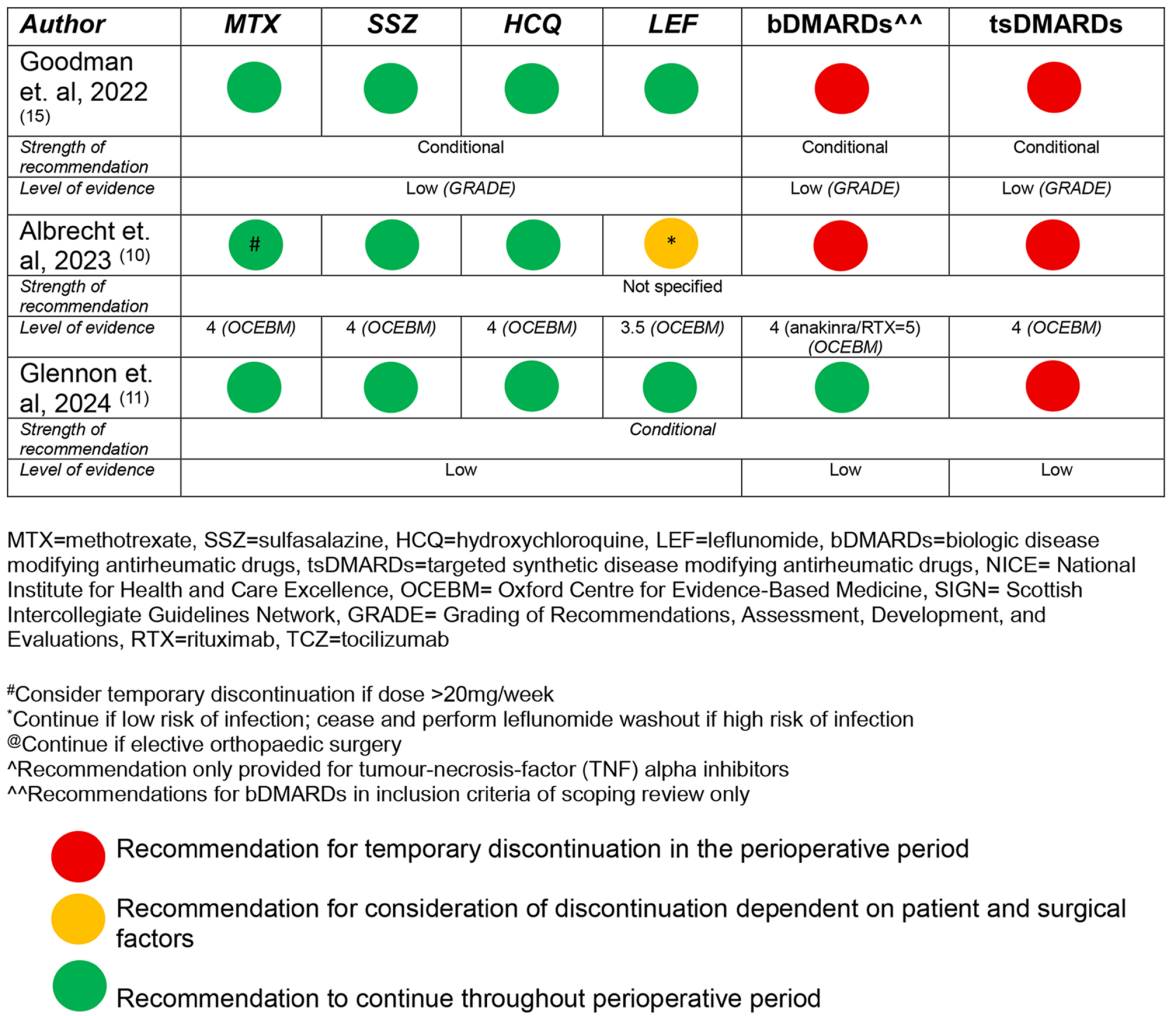
continued

### Recommendations regarding biological DMARDs (bDMARDs)

A recommendation regarding the perioperative use of bDMARDs was included in 8 guidelines, encompassing a broad range of agents, including TNF inhibitors, and non-TNF agents such as abatacept, tocilizumab and anakinra [10–12, 15, 16, 18, 20, 21]. A recommendation for rituximab was included in 5 guidelines [10–12, 15, 16]. The strength of the recommendation was weak/conditional in 5 (63%) [11, 12, 15, 18, 21], unspecified in 2 (25%) [10, 16] and cited as ‘moderate support’ in 1 (13%) [12]. All of the recommendations were based on low certainty evidence from case series or other observational studies. The level of agreement between panel members was high (70–100%) when reported in 3 of the 8 guidelines [10, 18, 20]. Temporary discontinuation of bDMARDs was conditionally recommended in 6 (75%) [10, 12, 15, 18, 20, 21], though patient and surgical factors were cited in all guidelines with regard to decision making. In the remaining two guidelines, a conditional recommendation against temporary discontinuation was provided in one [11], and a conditional recommendation for surgical timing with regard to the dosing cycle, along with a risk assessment of infection or flare of disease was provided in the other [16]. Recommendations for timing of surgery after temporary discontinuation of bDMARDs were highly variable, including the following: ‘3–5 half lives’, ‘discontinue for 2 weeks before or after surgery’, ‘1–2 months before surgery anti-TNF should be discontinued’, ‘time surgery at the end of dosing cycle’, or ‘discontinue one dosing cycle prior’. For rituximab, the recommendation for surgical timing ranged from 3 to 7 months following the most recent dose in 4 of the guidelines [10, 11, 15, 16]), with the final guideline stating that the timing of surgery should be ‘…defined more by the amount of B cells and in situations where the disease is well controlled’ [12]. Recommendations for the recommencement of bDMARDs following surgery was generally based on wound healing and absence of infection, with 3 (38%) conditionally recommending recommencement 14 days post-operatively, though the evidence for this recommendation was noted to be of very low quality. A temporal relationship with regards to the recommendations for continuation of bDMARDs was also noted, with the most recently published guideline suggesting continuation during the perioperative period [11]. A summary of the recommendations for the use of biological DMARDs in the perioperative period is included in Table 2.

### Recommendations regarding targeted synthetic DMARDs (tsDMARDs)

Of the 5 guidelines with inclusion of a recommendation for tsDMARDs [10, 11, 15, 18, 20], a conditional recommendation was provided in 4 (80%) guidelines [11, 15, 18, 20], with the strength of recommendation not specified in the remaining paper. Very low certainty evidence was available for formulation of the recommendations. The level of agreement was high (70–100%) when reported in 3 of the 5 guidelines [10, 18, 20]. As with the bDMARD recommendations, patient and surgical factors were considered to be important factors in decision-making regarding temporary discontinuation in the perioperative period. Specific recommendations for the timing of temporary discontinuation were present in 3 (60%) [10, 11, 15], ranging from 3 to 7 days prior to planned surgery, and recommencing 4–14 days post operatively. Wound healing and absence of infection were described as potential determinants when considering recommencement. A summary of the recommendations for the use of targeted synthetic DMARDs in the perioperative period is included in Table 2.

### Other recommendations for drugs outside of inclusion criteria

Recommendations for the use of any of mycophenolate mofetil, azathioprine, cyclosporine A and tacrolimus were present in 3 (35%) guidelines [10, 15, 19], based on low-certainty evidence and extrapolation of post-transplantation data in 1 guideline [19]. In addition, a single guideline included recommendations for belimumab, anifrolumab and voclosporin [15]. Recommendations were dependent on disease activity (‘non-severe’ or ‘severe’ systemic lupus erythematosus stratified in 1 guideline) [15]. A conditional recommendation for continuation of apremilast was present in a single guideline [10]. Guidance regarding the use of ixekizumab was present in 2 (17%) guidelines providing recommendations for rheumatic diseases other than RA [10, 15], both of which provided a conditional recommendation in favour of planning elective surgery at the end of the dosing cycle. A recommendation for ustekinumab was included in 2 guidelines [15, 16], suggesting consideration of surgery 1 week after the next scheduled dose of the agent. Recommendations for secukinumab and guselkumab were included in a single guideline [15], also suggesting consideration of surgery 1 week after the next scheduled dose of the biologic agent.

### Recommendations for the use of glucocorticoids

With regard to recommendations for glucocorticoids, included in 3 (25%) guidelines [10, 11, 19], temporary discontinuation was not recommended in any guideline. Two of the three guidelines recommended against stress dosing [10, 11] with no recommendation regarding stress dosing provided in the third [19].

### Limitations

The limitations of this review include a notable low quality evidence base, with a paucity of data for non-elective, non-orthopaedic surgery. Particularly for the newer DMARDs, there is a lack of evidence to guide recommendation formulation. In addition, there may be a potential benefit to broadening the search criteria to include guidelines published by other specialties (including Dermatology, Gastroenterology and Nephrology) and the target drugs, however applicability to AIRDs may still be restricted. Broadening the inclusion criteria to include drugs outside of the initial inclusion criteria (such as mycophenolate mofetil, azathioprine) would provide greater scope of recommendations but applicability to a non-transplant population may be limited. In addition, the inclusion of glucocorticoids as a target drug would broaden the scope of this review and enhance the clinical applicability.

## Discussion

Although guidelines for the use of DMARDs in the perioperative period are widely available, the content of published recommendations varies. This review has identified that current recommendations have been formulated based on evidence from trials involving patients undergoing elective total hip or knee replacement but there is little evidence to support recommendations in people undergoing other forms of surgery. A high level of agreement between panel members for the published recommendations was present in each included guideline. Whilst the included guidelines highlighted that patient factors (such as pre-operative AIRD control) and surgical factors (such as surgical site) should be considered in decision making regarding the perioperative use of DMARDs, no guideline provided specific recommendations regarding these elements.

All guidelines were published by national rheumatology societies from a range of countries however significant differences existed with regard to both the autoimmune rheumatic conditions and DMARDs included in each guideline. There was significant weighting towards the inclusion of RA in the majority of guidelines, affecting the generalisability of implementing these guidelines into clinical practice and the management of other autoimmune rheumatic diseases in the perioperative period.

Importantly, a patient representative was included on the minority of panels (*n* = 4). To our knowledge, there are no large studies assessing patient preferences regarding the management of their autoimmune rheumatic disease in the perioperative period. Publication of patient preferences from a small focus group (*n* = 12) in the United Kingdom suggested a willingness to accept the risk of infection associated with continuing DMARDs in the perioperative period to prevent a flare of inflammatory arthritis [22]. Incorporation of patient perspectives into formulation of recommendations may assist the panel’s ability to provide a balanced recommendation to clinicians and improve the implementation of these guidelines into clinical practice, particularly given the heterogeneity in clinical recommendations formulated by the individual clinical guidelines.

Recommendations between guidelines also varied, particularly those surrounding the recommendations for non-methotrexate csDMARDs, and for the timing of procedures in patients who are taking b/tsDMARDs. The limited evidence base for the recommendations, accompanied by differences in guideline content and a potential lack of generalisability limits implementation of these recommendations into practice. Interestingly, a temporal relationship was noted with regards to the recommendations for bDMARDs, with the most recently published guideline suggesting continuation of this class of drug during the perioperative period. This suggests that as evidence accrues over time, particularly with regards to newer DMARDs, this may be incorporated into future recommendations to aid clinical practice in this population.

In conclusion, the published recommendations for the perioperative use of DMARDs are highly variable, with the potential to unpredictably influence clinical practice. The evidence for these recommendations is generally of low quality, and limited to outcomes from studies assessing elective orthopaedic surgery, with a subsequent limitation of generalisability. As the newer classes of DMARDs are used over time, further evidence regarding their safety in this clinical period will likely be accrued, aiding development of clinical guidelines.

## Electronic supplementary material

Below is the link to the electronic supplementary material.

Supplementary Material 1

Supplementary Material 2

Supplementary Material 3

## Data Availability

All data generated or analysed during this study are included in this published article [and its supplementary information files].

## Abbreviations

RA: Rheumatoid arthritis
SpA: Spondyloarthritis
PsA: Psoriatic arthritis
AS: Ankylosing spondylitis
SLE: Systemic lupus erythematosus
JIA: Juvenile idiopathic arthritis
csDMARD: Conventional synthetic disease modifying anti-rheumatic drug
bDMARD: Biologic disease modifying anti-rheumatic drug
tsDMARD: Targeted synthetic disease modifying anti-rheumatic drug
AIRD: Autoimmune rheumatic diseases
GRADE: Grading of Recommendations, Assessment, Development and Evaluations
OCEBM: Oxford Centre for Evidence-Based Medicine
SIGN: Scottish Intercollegiate Guidelines Network
NICE: National Institute for Health and Care Excellence
