# Supplementary material for: Perioperative management with DMARDs in rheumatic diseases: a scoping review of clinical guidelines
Source: BMC Rheumatol. 2025 Jul 3;9:81. doi: 10.1186/s41927-025-00522-x (PMC12224603; doi:10.1186/s41927-025-00522-x)
Supplement: Supplementary file 1 — Supplementary Material 1 [file 41927_2025_522_MOESM1_ESM.docx]

**Supplementary file 1 – search strategies**

Ovid MEDLINE(R) ALL <1946 to April 17, 2024>

1     exp antirheumatic agent/      477149

2     (DMARD* or csDMARD* or bDMARD* or tsDMARD*).ti,ab.    7334

3     ((anti-rheumatic or antirheumatic) adj (drug* or agent*)).ti,ab.  10877

4     exp methotrexate/ or methotrexate.ti.     43835

5     pharmacological.ti,ab. or exp Janus Kinase Inhibitors/ or ((Janus adj kinase adj inhibitor*) or JAKi or JAK).ti,ab.     313110

6     1 or 2 or 3 or 4 or 5   784793

7     (guideline* or recommendation*).ti. 146442

8     (rheumat* or autoimmune or connective or arthritis).ti,ab.  519333

9     6 and 7 and 8     862

10    limit 9 to yr="2014-Current"  521

Embase <1974 to 2024 April 17>

1             exp antirheumatic agent/           1112969

2             (DMARD* or csDMARD* or bDMARD* or tsDMARD*).ti,ab.        23311

3             ((anti-rheumatic or antirheumatic) adj (drug* or agent*)).ti,ab.               18410

4             exp methotrexate/ or methotrexate.ti.  217562

5             pharmacological.ti,ab. or exp Janus Kinase Inhibitors/ or ((Janus adj kinase adj inhibitor*) or JAKi or JAK).ti,ab.           454885

6             1 or 2 or 3 or 4 or 5          1528852

7             (rheumat* or autoimmune or connective or arthritis).ti,ab.       757360

8             (guideline* or recommendation*).ti.     191757

9             6 and 7 and 8    1631

10           limit 9 to yr="2014-Current"      1061

11           limit 10 to conference abstract                271

12           10 not 11             790
