# Supplementary material for: Perioperative management with DMARDs in rheumatic diseases: a scoping review of clinical guidelines
Source: BMC Rheumatol. 2025 Jul 3;9:81. doi: 10.1186/s41927-025-00522-x (PMC12224603; doi:10.1186/s41927-025-00522-x)
Supplement: Supplementary file 2 — Supplementary Material 2 [file 41927_2025_522_MOESM2_ESM.docx]

Supplementary file 2

List of pre-defined categories for data extraction

Demographics:

- Author(s)
- Year of publication
- Title
- Country of development
- Region of development
- Rheumatic society developing guideline
- Standalone perioperative guideline or included as in a broader guideline for treatment of AIRDs?
- Included rheumatic conditions

Development of recommendations:

- Funding source
- Method of formulation of recommendation
- Structure of panel
- Declaration and management of conflicts of interest
- System used to rate evidence

Recommendations:

- Inclusion of a recommendation for csDMARDs OR bDMARDs OR tsDMARDs?
  - Level of evidence
  - Certainty of evidence
  - Level of agreement
  - Strength of recommendation
- Inclusion of a recommendation for other drugs (outside inclusion criteria)?
  - Level of evidence
  - Certainty of evidence
  - Level of agreement
  - Strength of recommendation
- Inclusion of a recommendation for prednisolone (outside inclusion criteria)?
  - Level of evidence
  - Certainty of evidence
  - Level of agreement
  - Strength of recommendation

Specific DMARD recommendations:

- Methotrexate
- Sulfasalazine
- Hydroxychloroquine
- Leflunomide
- Adalimumab
- Etanercept
- Golimumab
- Infliximab
- Abatacept
- Certolizumab
- Rituximab
- Tocilizumab
- Anakinra
- Secukinumab
- Ustekinumab
- Ixekizumab
- Belimumab
- Tofacitinib
- Updacitinib
- Baricitinib
- Drugs outside inclusion criteria: glucocorticoids (including prednisolone), mycophenolate mofetil, azathioprine, cyclosporine A, tacrolimus
